# Metabolomic Variation in Sugarcane Maturation Under a Temperate Climate

**DOI:** 10.3390/metabo15080558

**Published:** 2025-08-20

**Authors:** Yasuhiro Date, Chiaki Ishikawa, Hiroshi Ono

**Affiliations:** 1 Kyushu-Okinawa Agricultural Research Center, National Agriculture and Food Research Organization, Koshi 861-1192, Japan; 2 Institute of Food Research, National Agriculture and Food Research Organization, Tsukuba 305-8642, Japan; ishikawa.chiaki315@naro.go.jp; 3 Research Center for Advanced Analysis, National Agriculture and Food Research Organization, Tsukuba 305-8642, Japan; ono.hiroshi843@naro.go.jp

**Keywords:** sugarcane juice, NMR, metabolomics, ripening, temperate zone, sucrose, glucose, fructose, 4-aminobutyric acid

## Abstract

Background: Metabolomics is a powerful tool used for the evaluation of sugarcane components which are key factors influencing its response to biotic and abiotic stresses. However, little is known about the compositional variability and diversity of the sugarcane juice metabolome under practical field conditions in temperate climates. Methods: In this study, we characterized metabolomic differences and variability in sugarcane juice components during the maturation stage of nine cultivars grown in a temperate climate in Japan using a nuclear magnetic resonance-based metabolomics approach, aiming to provide insights into genotype-dependent adaptability to environmental and climate changes. Results: Principal component analysis revealed distinct metabolic profiles based on cultivar and maturation level. Notably, sucrose levels increased from September to December accompanied by decreased glucose and fructose levels across all cultivars. Early-maturing cultivars had high sucrose content even with shorter growing periods, suggesting particular advantages for sugar production in temperate climates. Additionally, 4-aminobutyric acid accumulated in all cultivars as maturation progressed. On the other hand, *trans*-aconitic acid, choline, and branched-chain amino acids showed cultivar-dependent trends. In one example, choline concentrations increased significantly in specific cultivars during maturation. Conclusions: These findings support a deeper understanding of metabolic adaptation and may aid in identifying cultivars better suited to environmental fluctuations.

## 1. Introduction

Sugarcane is a major industrial crop, yielding around 2 billion tons of produce worldwide in recent years [1]. It is characterized by high sucrose accumulation in its stalks, making it commercially valuable for global sugar and bioethanol production. Sugarcane juice, extracted by compressing or squeezing the stalks, is an essential feedstock for manufactured products. The juice contains a wide range of metabolic components that play vital roles in product quality and function. Therefore, metabolomic characterization and evaluation of compositional variability and diversity in sugarcane juice are important for quality management and sustainable production of end products [2].

Metabolomics is a powerful analytical approach that enables comprehensive detection of diverse metabolites and the extraction of features from biological and ecological systems. It has also been used to characterize and evaluate the metabolites in sugarcane juice. For example, Ali et al. applied a multiplex metabolomics approach, including mass spectrometry and nuclear magnetic resonance (NMR) spectroscopy, to profile metabolites in sugarcane juice and molasses [3]. Casarotti used several analytical techniques, including mass spectrometry and NMR, to elucidate enzymatic browning mechanisms in sugarcane juice [4]. We also adopted an NMR-based metabolomics approach to assess compositional variability and stability in the sugarcane juice metabolome and to identify characteristic profiles associated with differences in harvest period [2]. Thus, the metabolomics approach has been effectively applied to sugarcane juice analysis, especially in research focusing on metabolites and their related reactions.

Sugarcane is primarily cultivated in tropical and subtropical zones, but it is also grown in near-temperate and temperate regions, such as Honshu in Japan [5], Louisiana in the USA [6], and Ziyuan County in Guangxi Province, China [7]. In temperate regions, sugarcane has a relatively short growing season, mainly from early spring to late fall, owing to freezing and frost damage occurring when the temperature decreases sufficiently [8]. Consequently, sugarcane cultivars for these regions have been developed with a focus on early maturation and cold tolerance. For instance, the cultivar Kurokaido, developed in Japan, exhibits early maturation and robust ratooning at low temperatures [9,10]; the cultivar NiTn18, also developed in Japan, shows strong growth and ratooning under mulch-free, low-temperature conditions [9,11]; and cultivars in Louisiana tend to mature early and tolerate cold better compared with those in other sugarcane-growing regions [8,12,13]. Additionally, the overwintering ability of sugarcane hybrids and relatives has been assessed in the northern Kanto region of Japan [14]. Cold resistance varies markedly by cultivar, prompting efforts to identify chemical markers for cold tolerance to expedite breeding [15]. Metabolomic composition is also cultivar-dependent [16], influencing not only cold resistance but also other abiotic stress responses critical to cultivar adaptability under changing environmental and climate conditions. However, limited information exists on the compositional variability and diversity of sugarcane juice metabolomes in terms of genotype-dependent patterns under practical field conditions in temperate climates.

The objective of this study was to characterize metabolomic differences and variability in sugarcane juice components at the maturation stage under practical field conditions in a temperate climate, aiming to gain insights into cultivar-dependent adaptability to environmental and climate changes. To this end, we employed an NMR-based metabolomics approach, with the advantages of high reproducibility and minimal sample preparation [17], with multivariate analysis to evaluate the juice metabolomes of nine sugarcane cultivars grown in an experimental field in Tsukuba city, located in the northern Kanto region of Japan.

## 2. Materials and Methods

### 2.1. Plant Material

Nine sugarcane cultivars were used in this study: Harunoogi [18], KTn03-54 [19], Kurokaido [20], NCo310, Ni22 [21], Ni27 [22], NiF8 [23], NiTn18 [24], and RK03-3010 [25]. Single-bud stalks were planted on 24 March 2023 at the NARO experimental field (36°01’ N, 140°05’ E) in Tsukuba, Ibaraki, Japan. The field measured 6.0 × 13.2 m (length × width), with a furrow width of 120 cm and plant spacing of 15 cm, arranged in a triplicate randomized block design. Mulching was applied until May to promote growth by increasing the soil temperature. The soil type was Kanto loam (volcanic ash soil), and chemical fertilizer (9.0 g N m^−2^, 9.0 g P_2_O_5_ m^−2^, and 9.0 g K_2_O m^−2^) was applied twice (once as a basal application and once as a top dressing on 1 June). Irrigation was provided as needed when water availability was insufficient. Stalks, excluding heads and leaves, were harvested during 25–27 September (Sep), 30 October–1 November (Oct–Nov), and 5–7 December (Dec). Mild and moderate damaged (broken or cracked) stalks were included in this study, with the rates of 21.7% for Harunoogi, 14.0% for KTn03-54, 8.4% for Kurokaido, 21.7% for NCo310, 5.8% for Ni22, 7.6% for Ni27, 14.3% for NiF8, 33.0% for NiTn18, and 5.6% for RK03-3010. In total, 628 sugarcane stalks were used for further analysis.

### 2.2. Sample Preparation

Before juicing, harvested stalks were weighed, measured for length and diameter, and counted for node number. All stalks were processed using a benchtop compact milling machine (Kibi Juicer, TM-120, Matsuo Co., Ltd., Kagoshima, Japan) following the manufacturer’s instructions. Bagasse weight was recorded to calculate juice extraction efficiency. The Brix and pH values of the juice were measured using a refractometer (RX-5000α, ATAGO Co., Ltd., Tokyo, Japan) and a portable pH meter (LAQUAact D-73, HORIBA, Ltd., Kyoto, Japan), respectively. Extracted juices were transferred to ice immediately and stored at −30 °C after measurements were completed.

### 2.3. Sample Pretreatments for NMR Measurements

Juice samples (*n* = 628) were pretreated for NMR analysis following an established method [2]. Frozen juices were thawed at room temperature, shaken at 95 °C and 1400 rpm for 10 min using a ThermoMixer comfort (Eppendorf GmbH, Hamburg, Germany), and centrifuged at 4 °C and 20,630× *g* for 10 min. The resulting supernatants were mixed with an NMR buffer containing 100 mM K_2_HPO_4_/KH_2_PO_4_ (pH 7.4), 1 mM sodium trimethylsilylpropanesulfonate (DSS)-*d*_6_ as an internal standard, and 10% deuterium oxide.

### 2.4. NMR Measurements

Metabolomic data were acquired using a 700 MHz NMR spectrometer (Bruker Avance III HD with a 5 mm Cryo TCI probe, Bruker BioSpin GmbH, Rheinstetten, Germany). *J*-resolved spectroscopy was performed using parameters reported in a previous study [2]. Spectral processing and numerical conversion were conducted using TopSpin version 4.1.4 (Bruker BioSpin GmbH, Rheinstetten, Germany) and rNMR software version 1.1.9 [26], respectively, with normalization to the DSS internal standard, in accordance with the abovementioned study [2]. DSS and solvent peaks were excluded from further analysis.

### 2.5. Data Analysis

The dataset was analyzed using the NARO NMR-based metabolomics research tool (NARO-NMRtool), developed in-house by integrating open-source programs [27]. The tool comprises five modules: (1) data import, (2) spectral processing, (3) multivariate analysis, (4) machine learning, and (5) peak annotation (Table 1). In (1) data import, loading and numerical conversion were performed using the “nmrglue” [28] and “pandas” [29] libraries. In (2) spectral processing, zero-filling, line broadening, phase correction, and baseline correction were performed using the “nmrglue” library, whereas reference calibration and field trimming were performed using the “numpy” library [30], spectral binning/bucketing was achieved using the “pandas” library, solvent peak removal and normalization were conducted using the “pynmranalysis” library (https://github.com/1feres1/pynmranalysis; accessed on 22 July 2024), and peak alignment was completed using the “*i*coshift” library [31]. In (3) multivariate analyses, hierarchical clustering was performed using the “scipy” library [32], nonhierarchical clustering was conducted using the “pyclustering” library [33], principal component analysis (PCA) was completed using the “scikit-learn” library [34], discriminant analysis was achieved using the “ropls” library [35], and correlation heatmaps were generated using the “pandas” library. In (4) machine learning, classification and regression were performed using random forest or support vector machines via the “scikit-learn” library. In (5) peak annotation, the automatic statistical identification in complex spectra (ASICS) method was applied [36,37]. Before conducting analysis in the NARO-NMRtool, the dataset was scaled to a mean of 0 and variance of 1. Significant differences among groups were calculated using the Steel–Dwass test (*** *p* < 0.001, ** *p* < 0.01, * *p* < 0.05).

## 3. Results

### 3.1. Growth Characteristics of Sugarcane

**Figure 1:**
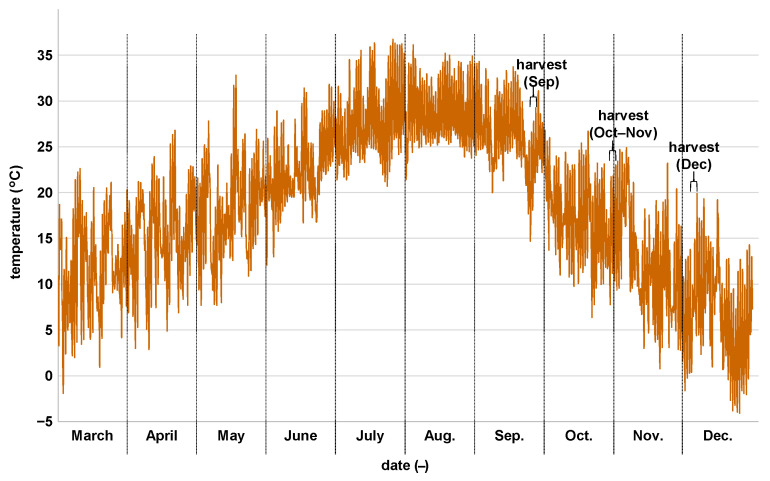
Air temperature from 1 March to 31 December 2023. Data retrieved from the Weather Data Acquisition System of Institute for Agro-Environmental Sciences, NARO (https://www.naro.affrc.go.jp/org/niaes/aws/) accessed on 18 October 2024.

Sugarcane was cultivated at the NARO experimental field in a temperate region, where air temperatures were ~0–35 °C (Figure 1). The growth characteristics of the nine cultivars harvested during Sep, Oct–Nov, and Dec were evaluated by measuring key agronomic traits: stalk weight, length, and diameter, internode count, and stalk count (Table 2). The cultivars Harunoogi, Kurokaido, NCo310, and NiTn18 had relatively slender stalks and produced a large number of tillers, whereas KTn03-54, Ni27, NiF8, and RK03-3010 had thicker stalks and fewer tillers. Juice extraction efficiency was approximately 50–60%, with Harunoogi and KTn03-54 showing the lowest and highest efficiencies, respectively (Table 3). Brix values increased in all cultivars from Sep to Dec. Kurokaido showed the highest Brix value, with a mean of 17.6% in Dec. Juice pH was ~5.0–5.3, with no significant differences observed among cultivars.

### 3.2. Metabolic Profiles in Sugarcane Maturation

**Figure 2:**
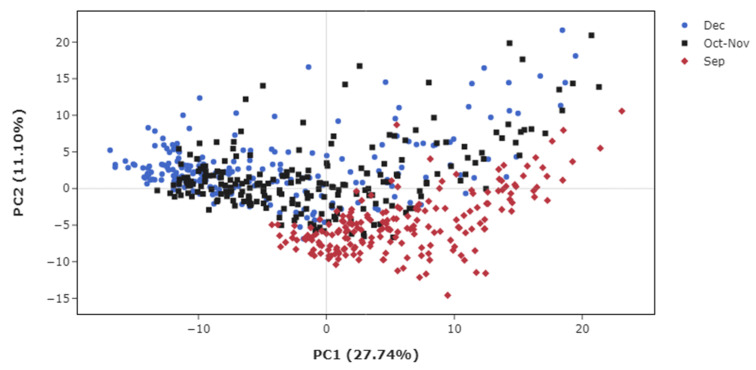
PCA of metabolomic variations in sugarcane juice across all samples (*n* = 628) from nine cultivars. Samples were collected during 25–27 September (red diamonds), 30 October to 1 November (black squares), and 5–7 December (blue circles).

**Figure 3:**
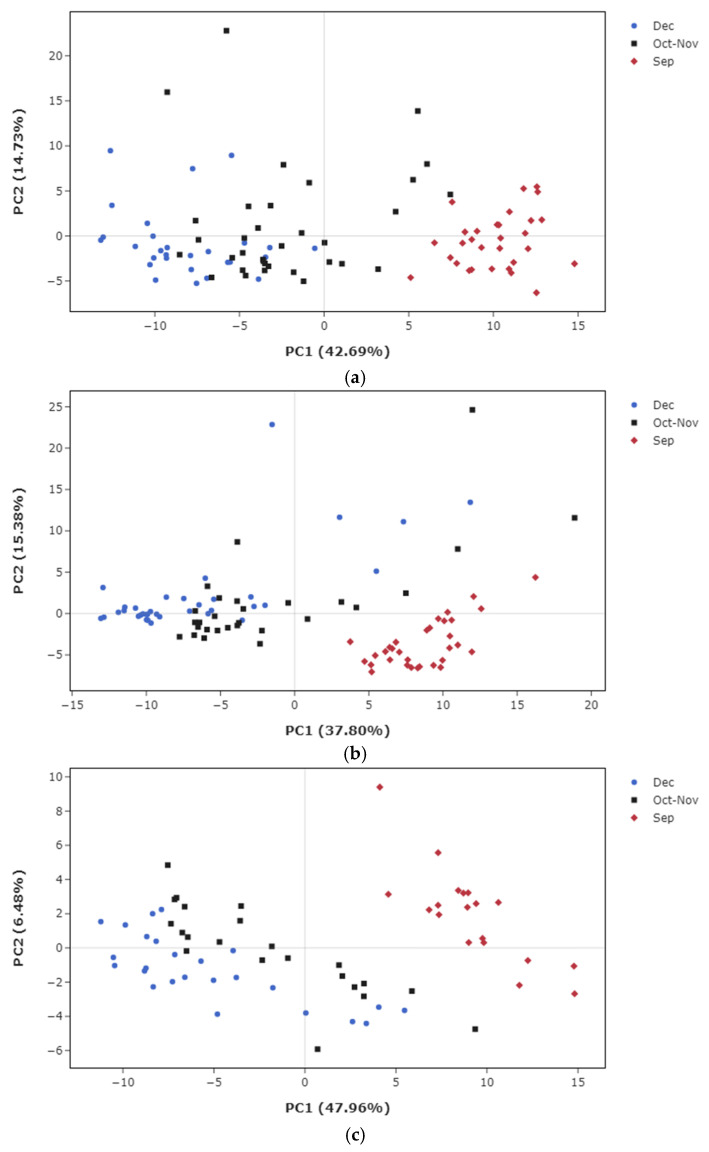
PCA of metabolomic variation in sugarcane juice within selected cultivars. Representative cultivars, namely Harunoogi (**a**), Kurokaido (**b**), and Ni27 (**c**), are displayed. Samples were collected during 25–27 September (red diamonds), 30 October to 1 November (black squares), and 5–7 December (blue circles).

An NMR-based metabolomics approach was used to analyze sugarcane juice and evaluate compositional transitions and diversity during maturation. PCA revealed distinct metabolic profiles by harvest period, reflecting the maturation stage (Figure 2 and Figure 3). Samples from Sep showed markedly different metabolic profiles compared with those from Oct–Nov and Dec, which were closely clustered (Figure 2). Removing cultivar effects further highlighted differences between the Oct–Nov and Dec samples (Figure 3).

### 3.3. Key Metabolites in Sugarcane Maturation

**Figure 4:**
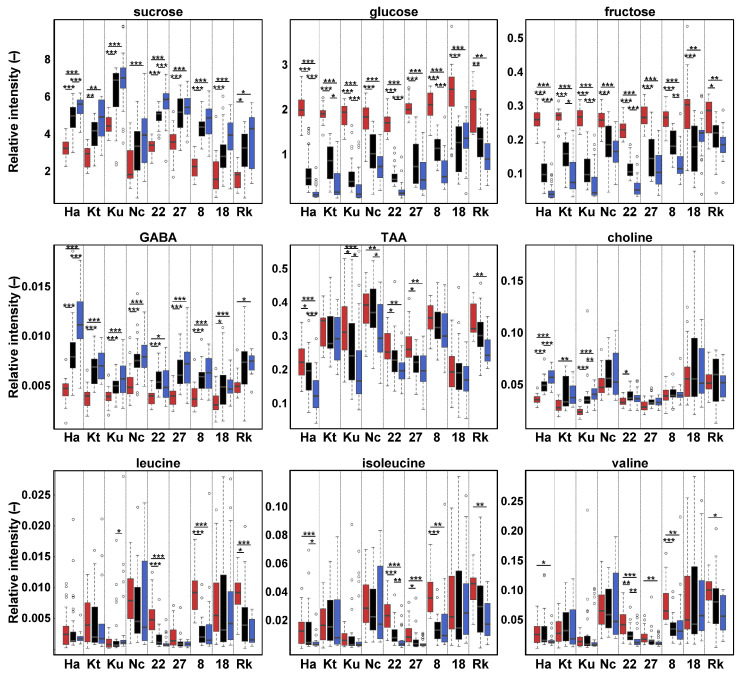
Temporal variation in key metabolites contributing to PCA clustering. Red, black, and blue represent samples for 25–27 September, 30 October–1 November, and 5–7 December, respectively. Relative intensity indicates an NMR signal intensity based on the DSS internal standard. The Steel–Dwass test was used to assess significance among groups: *** *p* < 0.001, ** *p* < 0.01, * *p* < 0.05. Ha, Harunoogi; Kt, KTn03-54; Ku, Kurokaido; Nc, NCo310; 22, Ni22; 27, Ni27; 8, NiF8; 18, NiTn18; Rk, RK03-3010.

Metabolites contributing to PCA separation were identified, and their temporal trends were evaluated in each cultivar (Figure 4). Levels of sucrose, a major metabolite, increased in sugarcane juice from Sep to Dec in all cultivars, aligning with maturation. In contrast, glucose and fructose levels declined. Additionally, 4-aminobutyric acid (GABA) concentrations were higher in sugarcane juice in Dec compared with Sep in all cultivars. Notably, these four metabolites exhibited consistent trends across cultivars. However, *trans*-aconitic acid (TAA), choline, leucine, isoleucine, and valine levels showed significant cultivar-dependent variation. TAA concentrations decreased significantly during maturation in Harunoogi, Kurokaido, NCo310, Ni22, Ni27, and RK03-3010. Choline concentrations increased significantly in Harunoogi, KTn03-54, Kurokaido, and Ni22 as maturation progressed. Leucine concentrations declined significantly with maturation in Ni22, NiF8, and RK03-3010 but increased significantly during November–December in Kurokaido. Isoleucine and valine concentrations decreased significantly during maturation in Harunoogi, Ni22, Ni27, NiF8, and RK03-3010.

## 4. Discussion

This study investigated the growth characteristics of nine sugarcane cultivars under a temperate climate, as well as compositional changes in their metabolomes during the maturation stage. Although most cultivars exhibited adequate growth in a temperate climate, several displayed shorter stalk lengths and lighter stalk weights. In particular, RK03-3010 appeared unable to express its full growth potential. Given that RK03-3010 was developed for Hateruma Island, located in the southernmost part of Japan [41], it is likely that the temperate climatic conditions at the study site were suboptimal for this cultivar. Additionally, considerable within-cultivar variability in stalk weight and length seemed to result from sugarcane lodging. Notably, NiTn18 had a high damaged rate (33.0%) due to broken and cracked stalks caused by lodging. NiTn18 is characterized by curved stalks and weak lodging resistance [11], which likely contributed to these results. Lodging not only increases damaged rates but also inhibits the growth of both affected and neighboring plants. This issue may be mitigated through improved cultivation practices, such as installing support structures.

Juice extraction efficiency varied across cultivars and is generally known to correlate negatively with fiber content in the sugarcane stalk [42,43]. This aligns with previous findings showing that Harunoogi contains more fiber in the stalk compared with that in NiF8 and Ni22 stalks [44], which is consistent with the present observation that Harunoogi displays the lowest juice extraction efficiency. Thus, cultivars with higher fiber content tend to yield less juice, and vice versa.

Brix values, mainly reflecting sucrose concentration, increased over time in all cultivars during maturation. The rise in sucrose levels was accompanied by lowered glucose and fructose concentrations, consistent with established metabolic processes converting glucose and fructose into sucrose in sugarcane via enzymes such as sucrose phosphate synthase and sucrose synthase [45,46]. These results indicate that the ripening process was successfully initiated and progressed in all cultivars, even under temperate conditions. However, the final Brix values and sucrose concentrations in Dec varied by cultivar. Kurokaido had the highest values, followed by Harunoogi, Ni22, and Ni27. Kurokaido is known for its ability to accumulate sucrose rapidly in its stalks and for reaching levels suitable for brown sugar production by the harvest period (typically November–December) in temperate regions of Japan [10,47]. In this study, the harvest had to be completed before the onset of frost; thus, the growing period was shorter than that in tropical or subtropical regions. Consequently, most cultivars likely reached harvest before fully maximizing sugar accumulation. Therefore, early-maturing cultivars, such as Kurokaido and Harunoogi, which exhibit high sucrose content even with shorter growing periods, may be particularly advantageous for sugar production in temperate climates.

GABA, a bioactive compound with known health benefits [48,49], is present at low concentrations in sugarcane juice [16]. Interestingly, GABA levels were significantly increased in Dec compared with Sep across cultivars. In plants, GABA accumulation is a known response to various stressors, including cold [50]. Therefore, the observed increase may reflect a stress response to low temperatures in the late growing season. Although further research is needed to elucidate the mechanisms and conditions underlying this accumulation, GABA has potential value in the development of functional sugarcane-based products.

Other metabolites, including TAA, choline, and branched-chain amino acids, showed cultivar-dependent variation during maturation. Each cultivar exhibits unique metabolic traits [16], and their metabolic responses to environmental stimuli are complex. These differences likely reflect intrinsic physiological properties and differential adaptability to environmental and climate changes. For instance, cultivars are known to vary in their cold tolerance, necessitating the use of multiple biomarkers to evaluate their hardiness [7]. From this perspective, the significant metabolites identified in this study, including sugars and GABA, could serve as valuable markers for assessing sugarcane maturation and stress responses under temperate field conditions. However, it remains unclear whether the observed metabolic variations are solely attributable to maturation, cold stress, or a combination of environmental and physiological factors. Continued accumulation of metabolomic data across a wider range of cultivars and conditions will deepen our understanding of genotype-dependent responses while supporting efforts in sugarcane breeding and quality management.

## 5. Conclusions

This study revealed metabolomic variations in nine cultivars during sugarcane maturation under temperate field conditions in Japan. Sucrose levels increased from Sep to Dec, accompanied by decreases in glucose and fructose concentrations across all cultivars. GABA accumulation was also observed consistently during maturation, whereas other metabolites, including TAA, choline, and branched-chain amino acids, showed cultivar-dependent trends. These findings suggest that sugarcane cultivars respond differently to environmental conditions, with implications for adaptability, ripening behavior, and product quality. Future work will focus on metabolomic analysis across diverse cultivars and environmental settings, which will provide valuable information for understanding physiological traits, supporting quality control in production, and guiding targeted sugarcane breeding programs.

## Figures and Tables

**Table 1:** Overview of modules, functions, and adjustable parameters in the NARO-NMRtool platform for NMR-based metabolomics analysis.

Module	Description/Parameter Options
**(1) Data import**	
Loading NMR data	Bruker fid data, Bruker spectra pdata, CSV data
Loading group data	CSV data
Loading objective variables	CSV data
**(2) Spectral processing**	
Zero filling (FID only)	Input a value (points)
Line broadening (FID only)	Input a value (Hz)
Phase correction (FID only)	Minimization around maximum peak, automated phase correction based on minimization of entropy [38]
Baseline correction	Distribution-based classification method [39]
Reference calibration	Select a reference peak range (ppm)
Field trimming	Select a range (ppm)
Binning/bucketing	Input binning/bucketing size (ppm)
Solvent peak removal	Select a range of solvent peaks (ppm)
Normalization	Reference peak, mean, probabilistic quotient normalization [40]
Peak alignment	*i*coshift algorithm
**(3) Multivariate analysis**	
Hierarchical cluster analysis	Method: ward, complete, average, etc. Metric: Euclidean, Bray–Curtis, cosine, etc.
Nonhierarchical cluster analysis	K-means, g-means, x-means
PCA	-
Discriminant analysis	Partial least squares, orthogonal partial least squares
Correlation heatmap	Pearson, Spearman, and Kendall correlations
**(4) Machine learning**	
Random forest	Classification, regression
Support vector machine	Classification, regression
**(5) Peak annotation**	
ASICS	-

**Table 2:** Agronomic characteristics of nine sugarcane cultivars at different harvest periods.

	Stalk Weight (g)	Stalk Length (cm)	Stalk Diameter (mm)	Internode Count	Stalk Count
	Mean ± S.D.	Mean ± S.D.	Mean ± S.D.	Mean ± S.D.	Total
**Harunoogi**	506.4 ± 205.4	176.6 ± 45.2	16.9 ± 2.3	10.8 ± 2.8	92
Sep	489.9 ± 170.9	170.9 ± 34.4	17.4 ± 1.9	10.6 ± 2.3	30
Oct–Nov	503.3 ± 224.8	170.0 ± 52.5	17.0 ± 2.2	10.3 ± 3.1	34
Dec	527.8 ± 212.6	190.8 ± 42.6	16.3 ± 2.7	11.6 ± 2.7	28
**KTn03-54**	575.7 ± 280.3	137.7 ± 57.8	21.4 ± 2.0	9.5 ± 4.1	43
Sep	646.1 ± 235.5	150.3 ± 44.5	22.1 ± 1.9	10.4 ± 3.2	13
Oct–Nov	534.9 ± 284.1	125.5 ± 58.9	21.5 ± 1.9	8.5 ± 4.2	16
Dec	557.0 ± 301.3	140.1 ± 64.3	20.6 ± 2.0	9.9 ± 4.6	14
**Kurokaido**	449.4 ± 182.2	153.3 ± 42.7	17.2 ± 1.9	8.1 ± 2.2	95
Sep	431.2 ± 147.9	148.1 ± 30.1	17.1 ± 1.8	8.0 ± 1.6	32
Oct–Nov	454.7 ± 191.5	148.2 ± 47.6	17.6 ± 1.6	7.8 ± 2.4	28
Dec	461.8 ± 200.6	162.2 ± 46.7	16.9 ± 2.1	8.5 ± 2.4	35
**NCo310**	398.3 ± 205.8	155.8 ± 60.8	16.1 ± 2.1	9.2 ± 3.5	83
Sep	365.9 ± 151.0	149.2 ± 45.3	16.0 ± 2.1	8.8 ± 2.6	26
Oct–Nov	416.4 ± 223.5	162.2 ± 67.1	16.0 ± 1.8	9.5 ± 3.8	29
Dec	409.8 ± 226.3	155.4 ± 65.6	16.4 ± 2.3	9.4 ± 3.8	28
**Ni22**	639.5 ± 220.4	200.6 ± 48.6	18.3 ± 1.6	10.6 ± 2.7	69
Sep	576.1 ± 208.4	180.2 ± 37.1	18.5 ± 1.7	9.4 ± 2.3	20
Oct–Nov	673.8 ± 201.5	204.8 ± 45.7	18.4 ± 1.6	10.8 ± 2.3	27
Dec	655.0 ± 240.3	213.9 ± 55.0	18.0 ± 1.5	11.5 ± 3.1	22
**Ni27**	897.7 ± 274.6	203.1 ± 37.7	21.9 ± 2.2	11.6 ± 2.5	66
Sep	855.2 ± 254.4	186.7 ± 41.4	22.8 ± 1.7	10.6 ± 2.7	19
Oct–Nov	872.3 ± 313.0	200.1 ± 38.5	21.6 ± 2.5	11.4 ± 2.4	22
Dec	952.4 ± 242.3	218.3 ± 26.3	21.6 ± 1.9	12.5 ± 2.0	25
**NiF8**	691.8 ± 356.3	166.2 ± 63.3	20.9 ± 3.3	9.3 ± 3.5	56
Sep	623.3 ± 370.9	147.6 ± 63.6	21.0 ± 4.5	8.5 ± 3.5	17
Oct–Nov	721.8 ± 339.8	171.5 ± 58.0	21.0 ± 2.3	9.3 ± 3.3	20
Dec	721.5 ± 351.5	177.4 ± 64.7	20.6 ± 2.9	10.2 ± 3.4	19
**NiTn18**	450.2 ± 266.4	150.4 ± 75.4	17.5 ± 2.0	9.7 ± 4.9	88
Sep	472.6 ± 229.2	155.8 ± 56.1	17.9 ± 1.7	9.8 ± 3.7	28
Oct–Nov	443.7 ± 297.9	144.9 ± 83.0	17.4 ± 2.1	9.5 ± 5.5	31
Dec	435.4 ± 262.6	150.9 ± 82.4	17.2 ± 2.1	9.8 ± 5.2	29
**RK03-3010**	450.7 ± 250.2	123.0 ± 59.7	20.5 ± 1.6	7.4 ± 3.3	36
Sep	361.7 ± 188.6	105.8 ± 48.7	19.9 ± 2.0	6.4 ± 2.6	13
Oct–Nov	421.4 ± 243.6	115.2 ± 58.8	20.5 ± 1.1	7.0 ± 3.2	13
Dec	604.4 ± 258.8	155.5 ± 61.2	21.4 ± 1.2	9.3 ± 3.3	10

**Table 3:** Juice extraction efficiency, Brix, and pH values of nine sugarcane cultivars at different harvest periods.

	Juice Extraction Efficiency (%)	Brix (%)	pH
	Mean ± S.D.	Mean ± S.D.	Mean ± S.D.
**Harunoogi**	51.1 ± 2.7	13.3 ± 2.2	5.20 ± 0.09
Sep	51.6 ± 2.2	10.8 ± 0.9	5.11 ± 0.04
Oct–Nov	50.8 ± 3.0	13.6 ± 1.4	5.21 ± 0.05
Dec	51.0 ± 2.7	15.5 ± 0.9	5.30 ± 0.04
**KTn03-54**	58.7 ± 2.7	11.6 ± 2.0	5.22 ± 0.10
Sep	58.4 ± 1.5	9.9 ± 1.2	5.11 ± 0.05
Oct–Nov	58.8 ± 3.1	12.0 ± 1.5	5.22 ± 0.08
Dec	58.8 ± 3.2	12.6 ± 2.1	5.31 ± 0.06
**Kurokaido**	55.4 ± 2.7	15.7 ± 2.8	5.15 ± 0.08
Sep	54.9 ± 2.3	13.2 ± 0.8	5.15 ± 0.09
Oct–Nov	55.2 ± 2.5	16.2 ± 2.7	5.13 ± 0.06
Dec	55.9 ± 3.1	17.6 ± 2.3	5.17 ± 0.07
**NCo310**	55.9 ± 3.3	10.2 ± 2.8	5.11 ± 0.13
Sep	55.0 ± 3.5	8.9 ± 1.8	5.00 ± 0.06
Oct–Nov	55.5 ± 3.0	10.1 ± 3.0	5.15 ± 0.12
Dec	57.3 ± 2.9	11.4 ± 2.8	5.19 ± 0.10
**Ni22**	55.2 ± 2.2	13.2 ± 2.1	5.22 ± 0.09
Sep	55.4 ± 2.0	10.7 ± 0.7	5.16 ± 0.06
Oct–Nov	54.8 ± 2.7	13.7 ± 0.8	5.23 ± 0.08
Dec	55.4 ± 1.8	15.0 ± 1.8	5.28 ± 0.07
**Ni27**	57.5 ± 2.1	13.6 ± 2.4	5.23 ± 0.10
Sep	57.7 ± 1.5	10.8 ± 1.4	5.13 ± 0.06
Oct–Nov	56.5 ± 2.3	14.5 ± 1.8	5.22 ± 0.09
Dec	58.2 ± 1.8	14.8 ± 1.6	5.32 ± 0.05
**NiF8**	55.6 ± 2.6	11.4 ± 2.4	5.13 ± 0.11
Sep	54.1 ± 2.8	8.8 ± 1.2	5.01 ± 0.03
Oct–Nov	56.6 ± 2.0	12.3 ± 1.1	5.14 ± 0.08
Dec	56.1 ± 2.3	12.9 ± 2.2	5.25 ± 0.04
**NiTn18**	56.7 ± 2.5	10.6 ± 2.6	5.19 ± 0.11
Sep	56.4 ± 1.7	8.7 ± 1.9	5.11 ± 0.06
Oct–Nov	56.6 ± 3.2	11.5 ± 2.1	5.19 ± 0.13
Dec	57.1 ± 2.3	11.6 ± 2.5	5.25 ± 0.07
**RK03-3010**	52.3 ± 2.5	9.4 ± 2.9	5.20 ± 0.12
Sep	50.9 ± 2.6	7.6 ± 1.3	5.08 ± 0.05
Oct–Nov	52.7 ± 2.4	9.8 ± 2.7	5.23 ± 0.10
Dec	53.5 ± 1.6	11.4 ± 3.1	5.31 ± 0.05

## Data Availability

The NMR data used in this study are available upon request from the corresponding author. The other data are included in this article.
